# King Edward's Hospital Fund for London

**Published:** 1910-01-08

**Authors:** 


### KING EDWARD'S HOSPITAL FUND FOR LONDON.

A meeting of the General Council of King Edward's Hospital Fund for London was held on the 3rd inst., at the Offices of the Fund, 7 Walbrook, E.C.

There were present Sir William Church (in the chair), the Rev. T. Bowman Stephenson, D.D., Lord Sandhurst, the President of the Royal College of Physicians (Sir R. Douglas Powell, Bart.), the Right Hon. Sir Savile Crossley, Bart., the Right Hon. Sir Joseph Dimsdale, Bart., the Right Hon. Sir Edgar Speyer, Bart., Lieut.-Col. Sir Arthur Bigge, Sir Horace Marshall, Sir John Craggs, Sir Cooper Perry, Dr. Edwin Freshfield, Mr. John G. Griffiths, Mr. Albert G. Sandeman.

A letter received by the Honorary Secretaries from Sir Arthur Bigge on behalf of His Royal Highness the President, was read as follows :—

"The Prince of Wales regrets that he is unable to be in London on January 3, and that consequently he cannot preside at the meeting of the Council. His Royal Highness desires to inform the Council that he has reappointed the Finance Committee as before constituted. Lord Richard Cavendish and Mr. Danvers Power retire from the Executive Committee, and the Hon. Sydney Holland, and Dr. J. R. Bradford, M.D., F.R.C.P., have been appointed, and the other members reappointed. To the Distribution Committee His Royal Highness appoints Mr. Danvers Power, Dr. F. H. Champneys, M.D., F.R.C.P., and Mr. Y. R. Eccles, and reappoints the other members except the Rev. Canon Barnett, who retires. Dr. J. Kingston Fowler, M.D., F.R.C.P., is appointed to the Convalescent Homes Committee, Mr. Alfred Willett, F.R.C.S., and Sir Richard Havelock-Charles retiring—the other members are re-appointed. His Royal Highness greatly regrets the loss of the valuable assistance of Mr. Willett, and is very sorry that Mr. Hugh Smith, who has been actively associated with the Fund since its foundation, is temporarily indisposed and unable to be present at Monday's meeting of the Council. His Royal Highness trusts that he may soon be restored to health."

The formal order signed by His Royal Highness, the President, appointing the members of the General Council, members of committees and officers, respectively, for the year 1910, was read. The resolutions providing for the work of the Fund for 1910, which were approved at the meeting of the President and General Council held at Marlborough House on December 13, 1909, were formally adopted.

